# Median Rhomboid Glossitis: A Developmental Disorder Involving the Central Part of the Tongue

**DOI:** 10.7759/cureus.48908

**Published:** 2023-11-16

**Authors:** Hussain Ali John, Kajal Ahuja, Rishika Dakhale, Khushi Heda, Shweta Sedani

**Affiliations:** 1 Oral and Maxillofacial Surgery, Sharad Pawar Dental College and Hospital, Datta Meghe Institute of Higher Education and Research, Wardha, IND; 2 Orthodontics and Dentofacial Orthopaedics, Sharad Pawar Dental College and Hospital, Datta Meghe Institute of Higher Education and Research, Wardha, IND; 3 Public Health Dentistry, Sharad Pawar Dental College and Hospital, Datta Meghe Institute of Higher Education and Research, Wardha, IND; 4 Conservative Dentistry and Endodontics, Sharad Pawar Dental College and Hospital, Datta Meghe Institute of Higher Education and Research, Wardha, IND

**Keywords:** tongue lesion, oral lesion, developmental disorder, papillary atrophy, median rhomboid glossitis

## Abstract

Median rhomboid glossitis (MRG), also known as posterior midline atrophic candidiasis, is a developmental disorder of the oral cavity. It usually involves the central part of the dorsum surface of the tongue. It is a papillary atrophy that is generally well-defined and symmetrical in presentation. It is usually a painless lesion, but some patients may complain of mild itching and irritation in that region. This lesion is more predominant in males than females. It has been widely argued that this lesion is not a developmental disorder but a clinical manifestation of a fungal etiology. This article presents a case report of a 55-year-old woman who reported to the oral medicine department in a tertiary care center in Wardha, India, with the chief complaint of an oval-shaped, discolored area on the dorsum of her tongue since birth. This article also emphasizes the role of a dental practitioner in diagnosing the lesion and appropriate patient education regarding the condition.

## Introduction

There are many developmental disorders of the tongue that result in its depapillation. Hence, it is very important that the practitioner who is diagnosing the condition has adequate knowledge about all the diseases and conditions that are related to the oral cavity [1,2]. Median rhomboid glossitis (MRG) is not a malignant lesion; in case of any doubt regarding its nature, a biopsy should be done after a round of antifungal therapy has been completed [1,3,4]. This is because, although the etiology of this lesion is an enigma, it is often associated with fungal infections like candidiasis [5]. Such lesions associated with a fungal etiology are more erythematous [1]. Other etiological factors like smoking, denture-wearing, and diabetes mellitus (DM) have also been suggested [5,6]. MRG is usually seen along with another finding in the palatal region, known as the 'kissing lesion', which occurs due to inflammation [1,7]. The lesion is more common in males, with a predilection of 3:1. It has been seen that this lesion is often unnoticed by patients until middle age or even later. Even after visiting many clinicians, this disease might go undiagnosed. This emphasizes the need for accurate knowledge about MRG and related lesions amongst dentists and other healthcare professionals to diagnose this lesion at the very first visit of the patient [1].

## Case presentation

**Figure 1:**
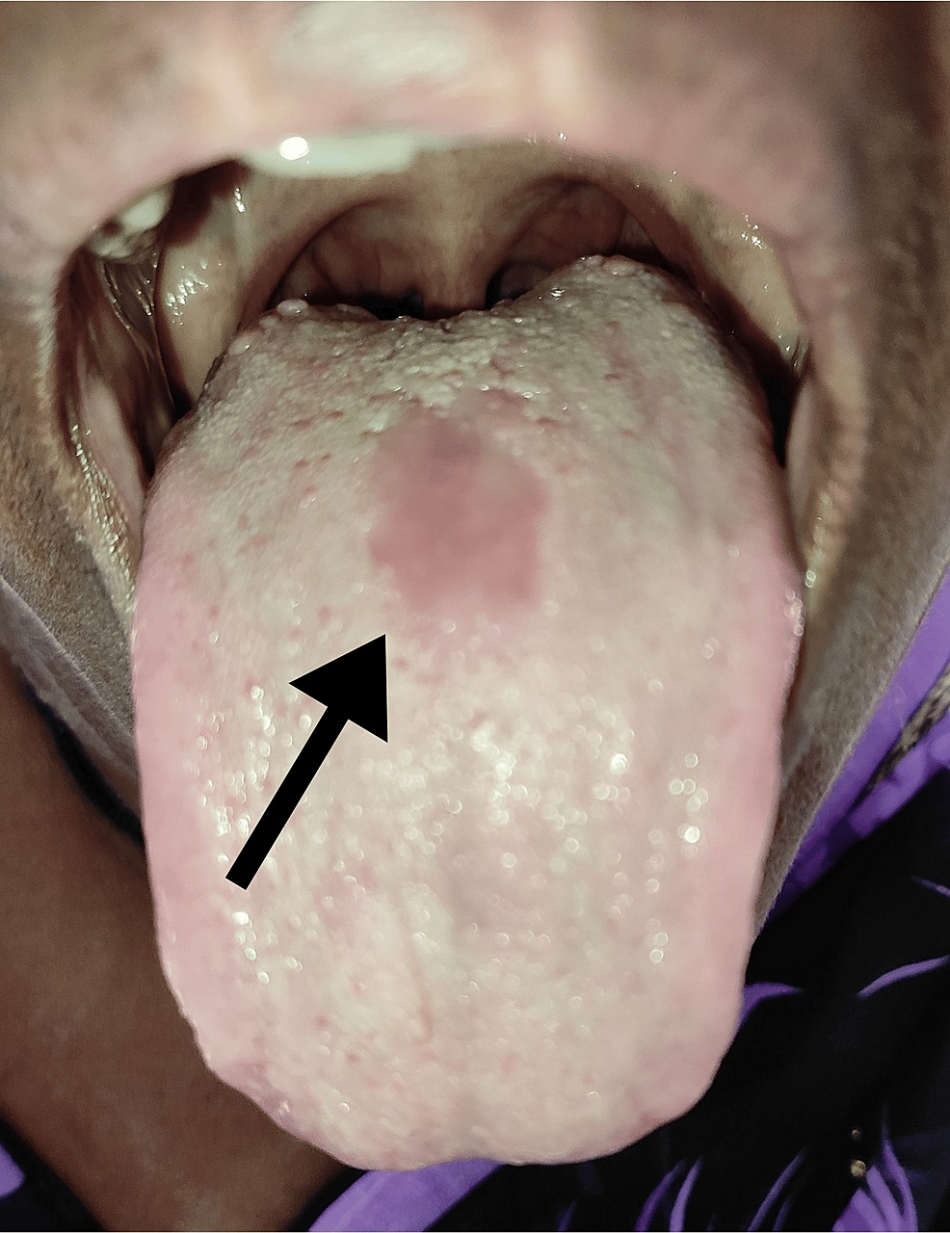
Intra-oral examination showing a roughly oval-shaped depapillated lesion on the dorsum of the tongue

A 55-year-old woman reported to the oral medicine outpatient department with the chief complaint of a patch of discoloration on the dorsum of her tongue since birth. The patient noticed an oval-shaped patch of pink at the center of her tongue when she was about 12 years old. She had previously visited three dentists and one dermatologist in the surrounding area, but they could not diagnose the condition. She was then referred to the current hospital. The lesion was asymptomatic, and no discomfort or irritation was reported. On intra-oral examination, a depapillated area was seen in the center of the dorsum of the tongue anterior to the circumvallate papilla of about 1 cm × 2 cm in size, roughly oval in shape; the surface was smooth; and the margins were well demarcated. On palpation, there was no local rise in temperature or tenderness present. Figure 1 shows the intra-oral examination of the patient.

On the basis of the history given by the patient and the clinical examination performed by the clinician, MRG was diagnosed. Based on the oral surgeon’s perspective and considering all the factors, the patient was advised that no invasive investigation like a biopsy should be done as the lesion is painless, long-standing, and not suspected of carcinoma. The patient was educated about the nature of the lesion, and no treatment was advised as the patient was asymptomatic.

## Discussion

Median rhomboid glossitis is a rare developmental condition of the tongue impacting 0.01-01% of the population [8]. It is more commonly seen in males than females. MRG is such a lesion that requires a decent degree of knowledge of oral medicine and dermatology to diagnose. The lesion's surface may be nodulated or smooth. It may be oval/elliptical, circular, or rhomboid in shape. This area of depapillation is almost always anterior to the circumvallate papilla on the dorsum of the tongue [1]. The lesion is more commonly seen in the mid-dorsal region and is usually less than 2 cm in its greatest dimension [1,9]. Although many etiological factors have been suggested for this condition, the most widely accepted is the fungal etiology. Candida albicans is said to be the causative agent. It is an opportunistic yeast that can cause various problems in immunosuppressive patients. Candidiasis is also a common infection in patients who are denture wearers [1,3,5]. Vigorous tooth brushing may also cause this lesion. According to a case report by Shindo T, a 57-year-old woman with no deleterious habits reported a chief complaint of a painful sensation and a lesion on the tongue. On investigation, she tested negative for candidal infection. The author concluded that she had median rhomboid glossitis due to robust tooth brushing, which resulted in the depapillation of her tongue. She was advised to stop brushing her tongue and was recalled for a follow-up. At the follow-up visit, which was one month later, the lesion and the patient's symptoms had improved [10]. The treatment of choice for symptomatic lesions is antifungal therapy, and it has been seen that some of the lesions completely disappear after it. Drugs like nystatin, fluconazole, miconazole, and clotrimazole are used. A biopsy can be performed after a round of antifungal therapy if the practitioner is unsure of the nature of the lesion. Usually, no treatment is necessary, but if the patient wants to get the lesion removed for aesthetic reasons, surgical removal can be done. Recurrence of the lesion after surgery is not expected [1,3]. Pyrosequencing is a new molecular technique for diagnosing candida-associated MRG. It is a real-time deoxyribonucleic acid (DNA) sequencing method, which is more accurate than the currently available conventional modalities of investigation for this lesion [11].

## Conclusions

Median rhomboid glossitis (MRG) is an often unnoticed and asymptomatic developmental disorder of the tongue. This lesion often goes undiagnosed up until the middle ages of the person; decent knowledge regarding diseases of the tongue is needed to effectively come to a diagnosis; hence, the role of healthcare professionals like dentists is important as they are the ones who primarily deal with the oral cavity and can diagnose the disease during the very first oral examination of the patient. If there is a dilemma regarding the nature of the lesion, a biopsy should be advised. If lesions are symptomatic, then antifungal therapy is advised, but in cases where the lesion is asymptomatic, no treatment is necessary.
